# Emergent Meningoencephalitis Following Spinal Anesthesia: A Case Report

**DOI:** 10.1155/crdi/9468161

**Published:** 2025-06-08

**Authors:** Yara Mouawad, Mahmoud El-Hussein, Joelle Kalaji, Patrick Nasrallah, Cima Hamieh, Eric Revue

**Affiliations:** ^1^Lebanese American University Medical Center, Beirut, Lebanon; ^2^Emergency Medicine Department, APHP–Lariboisiere Hospital, Paris, France; ^3^Geriatrics Department, Groupe Hospitalier Intercommunal Le Raincy, Montfermeil, France

## Abstract

**Background:** Iatrogenic meningitis is a rare but increasingly reported condition, particularly following invasive spinal procedures. The incidence is uncertain, ranging from none to two cases per 10,000 operations. Most cases involve infections with viridans streptococci. Septic meningitis presents with neurologic symptoms and elevated white blood cell counts in the cerebrospinal fluid (CSF), potentially leading to significant long-term neurologic damage or death if not promptly treated.

**Case Presentation:** A 32-year-old previously healthy male presented with severe headache, phonophobia, photophobia, nuchal rigidity, and fever, one day after undergoing anterior cruciate ligament (ACL) reconstructive surgery under spinal anesthesia. Initial evaluation revealed a Glasgow Coma Scale (GCS) score of 11, leukocytosis with neutrophilia, and turbid CSF with 3200 WBC/mm^2^, 100% neutrophils, and Gram-positive diplococci identified *as Streptococcus salivarius.* Blood cultures confirmed the same pathogen. The patient was diagnosed with meningoencephalitis, likely contracted via the spinal anesthesia procedure. Despite initial deterioration requiring intubation and intensive care, the patient responded well to targeted antibiotic therapy and was discharged in stable condition.

**Discussion:** The clinical outcome of iatrogenic bacterial meningitis significantly depends on the virulence of the infecting organism. High-virulence pathogens have been associated with higher mortality rates, whereas infections caused by low-virulence bacteria like viridans streptococci generally have a more favorable prognosis. The infection likely occurred due to contamination during the spinal anesthesia procedure, despite the use of standard aseptic precautions. This underscores the importance of stringent infection control measures, including the use of face masks, thorough handwashing, sterile gloves, and appropriate skin antisepsis.

**Conclusion:** This case highlights the potential for meningoencephalitis following spinal anesthesia, a rare but serious complication. Adhering to stringent aseptic guidelines and protocols is critical to prevent such infections. Prompt diagnosis and treatment are essential to mitigate the risks of severe neurologic damage and improve patient outcomes. Further research is needed to better understand the infection control measures that can prevent iatrogenic meningitis in spinal procedures.

## 1. Introduction

Despite iatrogenic meningitis being a rare condition, case reports are becoming more common in the literature [1, 2]. The frequency of this disease is uncertain, with reports ranging from nil to two cases per 10,000 operations [3–5]. Most reported cases are associated with medically invasive spinal operations, complicated by infections, with viridans streptococci often identified as the causal agent [2, 6, 7].

Septic meningitis is defined as the presence of neurologic signs and symptoms and elevated white blood cell count in cerebral spinal fluid, with the predominance of polymorph nuclear cells with or without positive CSF culture [8]. Depending on the virulence of the bacteria, the patient's comorbidities, and the onset of diagnosis and treatment, septic meningitis can present a considerable risk of long-term neurologic damage and even death [9].

This article presents a case of meningoencephalitis as a complication of spinal anesthesia used during an anterior cruciate ligament (ACL) reconstructive surgery.

## 2. Case Presentation

A 32-year-old previously healthy male presented to the emergency room with a one-day history of severe headache, phonophobia, photophobia, nuchal rigidity, and fever. He is a nonsmoker, nonalcoholic, and has no known allergies. One day prior to his presentation, he had undergone reconstructive ACL surgery under spinal anesthesia and was discharged home the same day on low molecular weight heparin, anti-inflammatories, and paracetamol, with no initial complaints.

Upon arrival at the emergency room, the patient was febrile (38.2°C), and tachycardic (118 bpm), but with otherwise stable vital signs. His initial physical examination showed he was awake, alert, cooperative, and oriented with a Glasgow Coma Scale (GCS) score of 15. However, he quickly deteriorated, becoming agitated, confused, and aphasic, with his GCS dropping to 11. His surgical wound was clean with no signs of infection, and there was no apparent infectious focus, including no evidence of dental or periodontal infection upon oral cavity examination.

**Figure 1:**
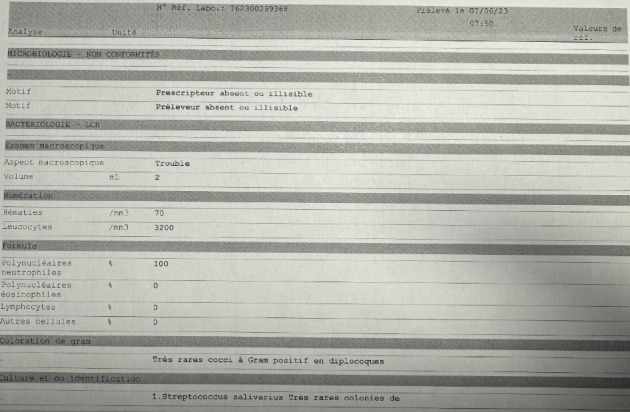
Initial CSF analysis and culture.

Laboratory tests, blood cultures, lumbar puncture, and imaging were conducted. An arterial blood gas analysis indicated metabolic acidosis with a pH of 7.315, a PCO2 of 32.9 mmHg, a PO2 of 84.7 mmHg, an HCO3 of 17.8 mmol/L, and a lactate level of 9.9 mmol/L. Leukocytosis and neutrophilia were evident with a CRP of 5 mg/L. The lumbar puncture revealed turbid cerebrospinal fluid (CSF) with 3200 WBC/mm^2^, all neutrophils (Figure 1).

The patient was immediately administered antibiotics (cefotaxime), antivirals (acyclovir), and corticosteroids to cover for possible meningitis or encephalitis.

Computed tomography (CT) scans of the chest and brain were normal, ruling out infectious processes and intracranial anomalies, respectively.

In CSF culture Gram-positive diplococci (*Streptococcus salivarius*) were seen (Figure 1), and a negative PCR multiplex. Blood cultures were also positive for *Streptococcus salivarius*. The admitting diagnosis was therefore meningoencephalitis, likely contracted through spinal anesthesia as the only recent invasive procedure.

**Figure 2:**
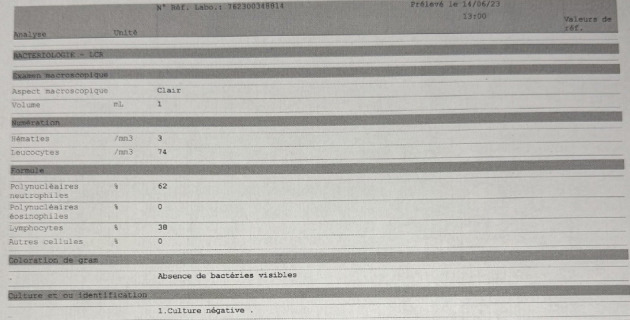
CSF analysis and culture after antibiotic treatment.

During his hospital stay, the patient's level of consciousness deteriorated, necessitating endotracheal intubation. He was admitted to the Intensive Care Unit (ICU) and continued on cefotaxime at 300 mg/kg/day, with other treatments discontinued. After two days, the patient showed clinical improvement and was extubated. He was transferred to the general ward after 5 days on the same treatment regimen. After 1 week of antibiotherapy, the patient had sterile CSF cultures with CSF analysis showing 74 WBC/mm^2^, and 62% neutrophils (Figure 2). The patient was switched to cefriaxone 3 g every 12 h before discharge to facilitate the antibiotherapy course at home for a total of 10 days. After 1-month follow-up was done and the patient was healthy with no sequelae.

## 3. Discussion

The virulence of the bacteria responsible for iatrogenic bacterial meningitis significantly impacts clinical outcomes [10]. In the literature, a 36% mortality rate was observed, associated with highly virulent pathogens such as Aspergillus spp. and *Staphylococcus aureus* [11]. In contrast, the prognosis for iatrogenic meningitis caused by low-virulence bacteria, such as viridans streptococci, has been favorable, with no fatalities reported [7].

The patient described in our case report had meningoencephalitis due *to Streptococcus salivarius*, a member of the viridans streptococci group. He experienced a favorable course with complete resolution.

Several processes contribute to the development of meningitis after lumbar puncture [10]. Previous literature stated that Infection can arise during bacteremia due to a “sudden drop in CSF pressure with a breakdown of the blood-brain barrier” [12]. Additionally, organisms may be introduced into the CSF space by the needle when passing through tiny capillaries during bacteremia [7, 12]. Since these stated hypotheses are highly unlikely and are not studied further research, the most probable cause is that the bacteria may be dispersed after skin disinfection onto the puncture area or equipment from the mouths of surrounding staff or the skin of the patient [13].

Aspirating CSF or allowing CSF to drip from the hub before injecting the anesthetic has been suggested as it may limit the risk of introducing infectious agents [2].

We conclude that extensive aseptic measures should be used to prevent lumbar puncture-associated meningitis [10]. Guidelines for spinal anesthesia should emphasize the need to use a surgical face mask with good bacterial filtering effectiveness to minimize bacterial contamination near the upper airway [14, 15]. Alterations to the mask should be avoided, and it should be replaced every 3 h or upon any contact. In addition to wearing the face mask, it is recommended to engage in comprehensive surgical handwashing and use sterile gloves. Performing diligent skin antisepsis, preferably twice, with an alcohol-based or iodine-based antiseptic that is compatible with the detergent used for the patient's skin preparation is also advisable [16].

## 4. Limitations

We lack enough data on the steps applied during the spinal anesthesia and the sterility of the products used, making us unable to link this complication to a particular stage in the procedure. We also did not test for the presence of *Streptococcus viridans* in any staff present during the surgery.

## 5. Conclusion

To sum up, the article discusses the case of a 32-year-old male who suffered unfortunate meningoencephalitis by *Streptococcus viridans* after spinal anesthesia. Although it is recognized in the literature that meningoencephalitis is a rare complication of spinal anesthesia, it can be prevented by adhering to guidelines and safe protocols. Early diagnosis and prompt management of bacterial meningoencephalitis is crucial as it can have detrimental effects on the patient.

## Data Availability

Data sharing is not applicable to this article as no datasets were generated or analyzed during the current study.
